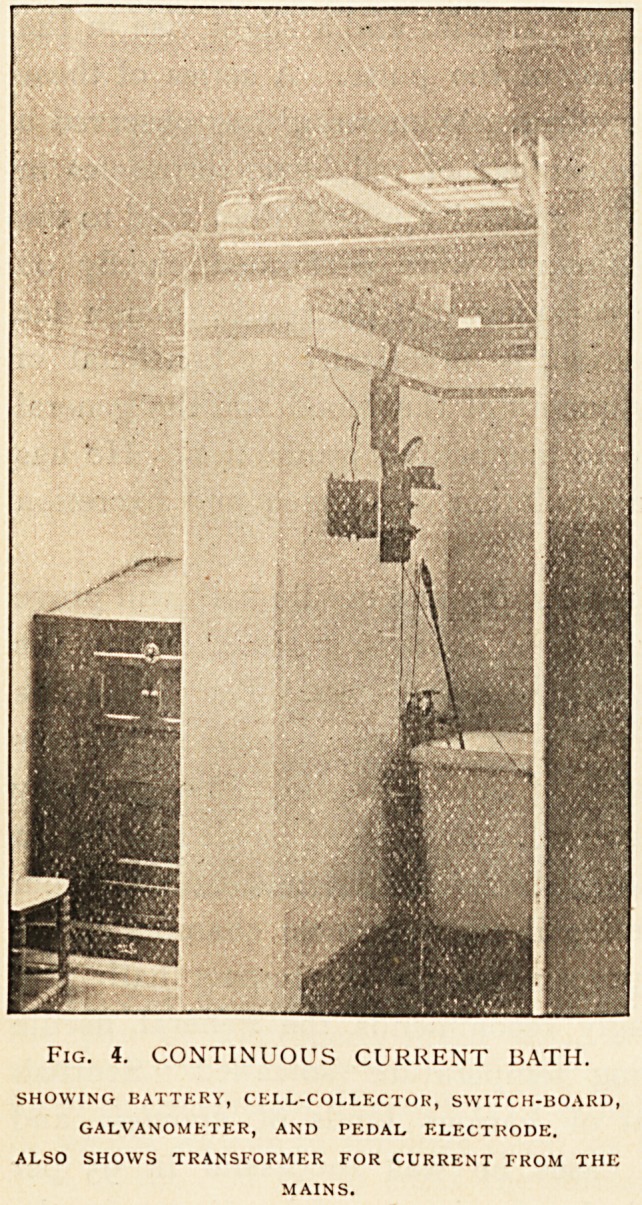# Notes on Some Thermal, Hydro-Thermal, Electric and Hydro-Electric Procedures, and the Indications for Their Use

**Published:** 1900-03

**Authors:** C. J. Whitby

**Affiliations:** Resident Physician, Clifton Grand Spa


The Bristol

Medico-Chirurgical Journal.

MARCH, 1900.

### NOTES ON SOME THERMAL, HYDRO-THERMAL, ELECTRIC AND HYDRO-ELECTRIC PROCEDURES, AND THE INDICATIONS FOR THEIR USE.

BY

C. J. Whitby, B.A., M.D. Cantab.,
*Resident Physician, Clifton Grand Spa.*

The empirical use of thermal and allied methods of treatment dates back to almost prehistoric times, but the rationale of their operation has been greatly elucidated by modern researches. In the Prussian Universities and Medical Schools considerable stress is now laid upon the teaching of this branch of therapeutics. Thus the addition was recently announced of a complete hydrotherapeutic and kinetic installation to the equipment of the Charité Hospital. By the systematic and graduated application of definite and appropriate stimuli (thermal, mechanical, electric) to the entire body-surface, or to particular parts of it, we can produce the following results: (1) The increased oxidation, and (2) the elimination of toxic products; (3) the stimulation of anabolic, or (4) of catabolic molecular activities; (5) the restoration of impaired or dormant functions; and (6) the establishment of a heightened condition of general nervous and vascular tone, where these were previously defective. Far too much stress is laid upon the mere eliminatory effect of these methods of treatment. It is to their great value as a species of organic or molecular *exercises*, capable of gradually arousing the latent powers of the organism, that the writer desires to call special attention. Thus, in a case recently observed, the manifestations of a general rheumatic poisoning were first distinctly aggravated, but ultimately dispelled, by the persevering use of a simple graduated needle-bath. It is hoped that the following paper may be of service to those who, while desirous of testing the value of physical methods, are deterred by the inaccessibility of technical information.

### I. Thermal.

(*a*) The Turkish bath, comprising three heated chambers, viz.:

i. A warm room, at a temperature of 115° F. to 130° F.
ii. A hot room, at a temperature of 130° F. to 150° F.
iii. A flue room, at a temperature of 160° F. to 200° F.

*Methods of using the Turkish Bath.*

(1) Full bath: Go straight into the flue room (iii.) and remain till slight oppression is felt; the hot room (ii.), next by the same rule. Remain thereafter in the warm room (i.) until called, the whole occupying 40 to 60 mins.
(2) Ordinary bath: The flue room first, for 6 mins.; the hot room next, for 15 mins.; and then the warm room, for 10 mins., more or less.
(3) Brief bath: The hot room (ii.) first, for 10 mins., and the warm room (i.) for 10 mins.
(4) Mild bath: The warm room (i.) for 20 mins.
(5) A warming merely: Five minutes spent in the warm room (i.) as a preliminary to some other bath.

On entering the bath the patient should wet the hair thoroughly, and then the attendant binds a triangularly-folded wet cloth turban-wise around the head. In cases of vaso-motor irritability or plethora, to obviate a rush of blood to the head, the feet may be kept in hot mustard and water. Syncope may be averted by applying cold wet cloths to the precordial region. Perspiration is encouraged by sipping cold water.

*Methods of concluding the bath.*

(1) By a general shampooing and soaping, followed by a spray or needle bath, and finally, in suitable cases, by a plunge bath.
(2) A hot soap-down without shampooing, followed by a needle or spray bath.
(3) Spray or needle bath only.
(4) In cases of cardiac or nervous debility, a mere tepid sponging may suffice.

(*b*) The Russian bath, consisting of a chamber kept at a temperature of 100° F., and further heated for use by steam to a temperature of 110° to 120° F. Generally speaking, the duration of the bath should be not less than 10 or more than 20 minutes. To conclude the process, precisely the same alternatives of shampooing, soaping, spray or needle bath, &c., are available as in the case of the Turkish bath.

FIG. 1. VAPOUR BATH.
SHOWING APERTURES FOR THE TREATMENT OF EXTREMITIES.

(*c*) The vapour or steam-box bath, general and local (Fig. 1).

i. *General.* The patient sits in a box so constructed as to allow the head to protrude. The box is heated in advance to 80° to 90° F. The patient sits with his feet in hot water and with a wet cloth bound round his head. Steam is allowed to flow freely into the box until slight oppression is felt and perspiration breaks out on the face, or until the temperature of the box is about 110° F. Thereafter it is partly or entirely turned off, but the temperature will be maintained for some time. The duration is 20 minutes, or less. Give cold water to drink as desired. Conclude with a spray or needle bath, or a simple sponge-down, as may be prescribed.

ii. *Local*. The hands, arms, feet, or legs may be inserted through apertures provided for the purpose into the interior of the vapour bath, and steamed for from 10 to 20 minutes. This is a very useful procedure in many cases of gout and rheumatoid arthritis.

(*d*) The radiant heat and light bath (Fig. 2). Dowsing's apparatus for the application of electrically-generated luminous heat to therapeutic purposes has been so widely discussed that a detailed description is unnecessary. The *general* bath consists of a set of four wedge-shaped copper reflectors, each containing

FIG. 2. RADIANT HEAT AND LIGHT BATH.
SHOWING COUCH FOR GENERAL BATH, AND AN APPLIANCE FOR LOCAL TREATMENT.

two long incandescent lamps of ground glass. These lamps are the source of the heat and light rays, and are so arranged that there are four of them on each side of the couch on which the patient lies. For a general bath the whole eight lamps are used simultaneously, but for local purposes any less number may be employed. There are also some special appliances for purely local use. The patient lies on the couch naked, except for a loose envelopment of lint soaked in tungstate of soda solution, and the whole apparatus is then covered in in such a way as to form a chamber, from which the head alone is excluded. The degree of heat is regulated with nicety by means of a rheostat controlling the lamps, and the temperature usually attained in giving a general bath is 350° F. Perspiration is very free as a rule. At the end of 35 to 40 minutes the patient is dried with a warm Turkish towel, and then rests until cool enough to dress. A sponge-down may be given if necessary, but in most cases is not considered advisable. For the radiant heat treatment of small parts of the body a much higher temperature (375° to 420° F.) can often be used with advantage. Of course, the sensations of the patient must be regarded to some extent, but I have never known the slightest burning or even scorching of the skin to be produced. There is a general consensus of opinion among those who habitually prescribe this form of treatment that in all, except perhaps traumatic, cases it is best to employ the full bath, *plus* some special concentration of heat upon the affected parts. In rheumatic and gouty affections a purely local treatment often tends to produce a mere metastatic shifting of the inflammation to some other locality.

*Physiological Effects.*—The effects of heat upon the organism, so far as they are known, may be briefly stated as follows:—(1) The temperature of the body is raised, the increase being *relatively* greater under the influence of moist than of dry heat. (2) Dilatation of the arterioles and capillaries of the skin, and consequent contraction of the deeper vessels, especially those of the splanchnic area; depletion of the abdominal viscera. (3) More or less acceleration of the heart's action, due in part to the diminished peripheral resistance. The excitant effect of dry heat upon the heart is relatively less than that of moist heat. (4) Diaphoresis and a fall in the mean arterial blood-pressure. (5) Increased respiratory activity, and increased oxidation of waste products, with consequent modification of their toxicity. (6) Increased renal activity, with a relative and absolute increase of the nitrogenous and other constituents of the urine; in other words, increased elimination of urea and uric acid.[1]

Where *pain* is present, as in cases of arthritis, neuritis,

[1] *Physiological and Therapeutic Effects of Transpiration Baths*, by Dr. A. Frey. Tr. by W. H. Gilbert, 1891. *J. Balneol. & Climatol.*, 1899, iii. 157, 229.

myalgia, one of the first effects of heat is to remove or diminish it—at least, in the great majority of cases. There can be little doubt that the artificial pyrexia produced by thermal treatment is beneficial to the constitutional cachexia which forms the basis of local inflammatory manifestations. An experiment of Penzo's is worth quoting in this connection. He injected into rabbits cultures of staphylococcus aureus and streptococcus, and made arrangements whereby one ear of the rabbit was subjected to a low and the other to a high temperature. In the ear which was subjected to a low temperature inflammatory reaction was retarded, but its course was unfavourably affected. In the other ear the reaction was quickened, but was less severe; there was less tendency to spread and a comparative absence of troublesome sequelæ.[1]

Some special effects of *luminous* heat have been described by several observers, and call for brief mention here. It is said that heat rays, *plus* light and actinic rays (as in the Dowsing bath) are more active and penetrating than dark heat, and Dr. R. T. Bowles suggests[2] that this may be due to the fact that the light rays are degraded into heat rays when they impinge upon the surface of the body, or as they pass through the tissues. Luminous heat has a much greater action upon the *skin* than dark heat, and the heat and light bath is certainly likely to prove of special benefit in chronic cutaneous diseases. Luminous heat is undoubtedly the most powerful diaphoretic we possess. In the Dowsing bath perspiration streams from the surface of the body, yet there is a marked absence of prostration or fatigue. Scientifically, there is, however, no such thing as a *hot-air bath.* Air acts to heat-rays as a vacuum, and the *air* of a Turkish bath should be kept as cool as is compatible with the comfort of the bather. It is a common error to suppose that such baths are heated by blasts of hot air (convection). A good Turkish bath is a *radiant* heat bath, but the heat rays are not to any considerable extent mixed with actinic and luminous rays.

*Indications.* The direct stimulant action of dry, and especi-

[1] *Arch. Ital. de Biol.*, 1897–98, xxviii. I.

[2] *J. Balneol. & Climatol.*, 1899, iii. 167.

ally of luminous, heat upon the body-surface, and the improved nutrition of the skin effected by a general determination of the blood to the cutaneous vessels and a general acceleration of its flow, indicates the radiant heat bath as an appropriate remedy for those forms of *skin disease* (*e.g.*, some cases of *psoriasis*, *dry eczema*, *lichen planus*, &c.) in which an atrophic or dystrophic, rather than an inflammatory, affection is to be combated. In more acute and irritable conditions the moist heat of the Russian bath (*b*), or the general vapour (steam-box) bath (*c*, 1), is preferable. When the extremities are affected general treatment may be augmented, or even replaced, by local steam baths (*c*, 2) or local applications of dry (radiant) heat. Krause reports the rapid cure of a psoriasis of the leg by such local treatment.[1]

The normal quantity of sweat is about half a litre per day, but may easily be increased four or six-fold. The extreme modifiability of this function indicates it as a proper field for therapeutic effort. By active diaphoresis considerable quantities of water are withdrawn from the blood circulating in the cutaneous vessels, and a temporary concentration of the blood is brought about. To restore the normal balance any excess of serous liquid which the tissues may contain will probably be in part re-absorbed into the circulation, for, as we know, the chemical composition of the blood is on the whole very uniform. Hence in the treatment of the serous effusions of *pleurisy*, *ascites*, *synovitis*, and *iritis*, the Turkish bath (*a*, 1, 2, or 3), or, better still, because of its greater diaphoretic power, the radiant heat bath (*d*), should, in the absence of decided contra-indications, find a place. Thermal treatment has even been suggested for early stages of ovarian cyst-formation.

The experiments of Arloing[2] and others prove that the *toxicity* of sweat is also very variable, and is increased by conditions (*e.g.* muscular effort) which raise the toxicity of the blood. Here then we have an indication for thermal diaphoresis in the treatment of *lithæmia*, "*suppressed gout*", *renal insufficiency*, *chronic Bright's disease*, and other forms of auto-

[1] *München. med. Wchnschr.*, 1898, xlv. 621.

[2] *Brit. M. J.*, 1897, ii. 678.

intoxication.[1] An advantage of the Turkish bath treatment of such conditions is that, while increasing the activity of the skin, it has no prejudicial effect upon the functions of the kidneys. The toxæmia of *epilepsy*[2] has been extensively treated by Cabitto[3] with sweating baths, with the result of a decided diminution in the frequency and severity of the fits.

The thermal treatment of *gout*, *rheumatism*, and *rheumatoid arthritis*, as well as of *gouty* and *rheumatic neuritis*, *myalgia*, &c., is so generally recognised that I will confine myself here to a few practical suggestions. For *acute gout*, for most cases of *sciatica* and *neuritis*, and for *rheumatoid arthritis*, the radiant heat bath is to be preferred as being (1) more powerful, yet (2) relatively less exhausting, than the other forms of thermal treatment. The Russian bath and the steam-box bath (general or local) have a very good effect in the treatment of the less severe manifestations of *gout*, but dry heat is as a rule preferable (*a*, 2 or 3) in *rheumatic* cases.

Cases complicated by *bronchitis* or *asthma* will generally do best with the Russian bath, because of the sedative and resorptive effect of the inhaled steam. Russian baths are also useful in cases of *chronic* or *acute nasal catarrh*, and, in one instance at least, *deafness* of several years' duration, due to Eustachian catarrh, was, to my knowledge, cured by a course of Russian baths.

Brief or mild Turkish baths (*a*, 3 or 4), followed by the spray bath alone, or by tepid sponging, are often extremely beneficial in the milder cases of *valvular disease* of the heart. The attendant should be warned by adding the words "*with care*" to the prescription, and any cardiac discomfort must be the signal for the termination of the bath. As a matter of fact, however, in ten years' experience, during which time I have treated many such cases, I have never seen any ill effects from their thermal treatment. The circulation is facilitated, almost

[1] Cf. Self-poisoning and its Treatment, by C. J. Whitby, M.D., *Bristol M.-Chir. J.*, 1896, xiv. 307.

[2] Cf. *Lancet*, 1899, i. 1241.

[3] *Riv. sper. di Freniat.*, 1897, xxiii. 36, 52; abstract in *Brit. M. J.*, 1898, Epitome, p. 11.

as much as by venesection, by the depletion of the congested viscera, and the appetite and general health are improved.

The common notion that Turkish baths necessarily lower the body weight is, of course, fallacious. Anabolic or katabolic effects may predominate according to the method employed. In cases of *neurasthenia*, *anorexia*, *anæmia*, and *convalescence*, I have often witnessed a gain in weight as the result of bi-weekly or tri-weekly *tonic* Turkish baths (*a*, 3 or 4), followed by a spray or needle bath only. In cases of *obesity* the bath should be longer (short of exhausting the patient however), and should be followed by a shampooing and, if possible, cold douching.

### II. Hydro-Thermal.

*Impact Baths:—*

i. *The spray bath.* Water is forcibly delivered horizontally through a rose (2½ in. diameter). The temperature is hot or warm to commence with, and is gradually lowered till cold or nearly cold. The patient meanwhile turns himself about, rubs the chest, back, &c., and occasionally brings the head under the spray. Usual duration, one to two minutes. The bath may be varied in many ways; *e.g.*, by alternately raising and lowering the temperature, by localising on certain organs or parts of the body, by lengthening the duration, or, by means of a reversible nozzle replacing the rose, may conclude with a general or local *cold douche.*

ii. *The needle bath* consists of two curved horizontal pipes connected by a number of smaller vertical pipes. These vertical pipes are perforated with small apertures at intervals of a few inches, the result being that many forcible jets of water inpinge simultaneously upon practically the whole body surface. The method of using the bath is the same as in the case of the spray bath, but a more stimulating effect is produced.

iii. *The douche* may be used as a general or local bath, and, as regards temperature, hot, cold, graduated, or alternating. Cold douches should be followed by friction until the circulation of the part is restored. In prescribing a douche the duration, temperature, &c., should always be definitely stated.

iv. *The Aix douche* (Fig. 3). This procedure is a combination of a hot or warm douche with systematic massage. The patient, undressed, seated on a short-backed wooden chair, has a fixed spray of water at 98° F. (or prescribed temperature) playing on the back and shoulders. Meanwhile the attendant massages in turn the feet, legs, thighs, hips, arms, back, chest, and abdomen, at the same time directing a stream of water at 98° F. (or prescribed temperature) from an open hose-pipe on the part just massaged. The joints are, in turn, flexed, extended, abducted, adducted, and rotated, and this is followed by (*a*) stroking with compression and (*b*) kneading of the joint and limb, and the next part is then taken in the same way. Average duration, 15 minutes, or as prescribed. Conclude with a brief general spray bath to cool down; rest for an hour after the bath.

FIG. 3. THE AIX DOUCHE.

v. *The ascending douche and spray.* The patient sits over the pan of a properly-fitted w.c., and water of any desired temperature is delivered vertically upwards with more or less force as prescribed, through (1) a ¼-inch nozzle, (2) a small rose, in such a way as to impinge upon, or even, if necessary, in the case of the douche, to enter the fundament. Duration, half to two minutes.

*Physiological Effects.*—A general impact bath stimulates the nervous system and brain more or less powerfully in proportion to the force, lowness of or abruptness of change in temperature, and form of distribution employed. A feeling of exhilaration is produced, which lasts for some time. Drs. Edgecombe and Bain[1] found that a strong needle bath, graduated from about 99° to 60° F., caused a decided quickening of the pulse, a rise

[1] *Lancet*, 1899, i. 1552.

in the arterial and a slight fall in the venous blood-pressure. These effects are attributed to (1) peripheral vascular constriction, (2) increased cardiac output. The effects of combined massage and warm douching (as in the Aix douche) are precisely the reverse; viz., a fall in the arterial and a slight rise in the venous pressure, with slowing of the pulse. A series of these baths produces a *cumulative* effect. Winternitz[1] has observed a decided increase in the percentage of red blood-corpuscles in the blood as the result of the general application of cold to the body surface. His experiments were performed chiefly on anæmic subjects; and he considers that the result, though due merely to altered distribution, is practically as beneficial in improving the respiratory quality of the blood and the general nutrition as if it were due to augmented production. He has also noted an increased assimilation of oxygen and excretion of carbonic acid.

*Indications.*—The graduated spray and needle bath will prove beneficial in cases of *neurasthenia*, *chlorosis*, *anorexia*, and in the secondary anæmia and debility of *convalescence*. They should be taken at least three times a week, and the frequency, lowness of temperature, and duration of the baths should be gradually increased as recovery progresses. Persons of feeble circulation with a tendency to cyanosis should be directed to take from three to five minutes warming in the outermost chamber of the Turkish bath (*a*, 5) as a preparation for the impact bath prescribed. Buxbaum strongly recommends the general needle bath of rapidly alternating temperature (so-called "Scottish douche") as a treatment of various kinds of *neuralgia* and *sciatica*. Of eighty-three cases he was successful in 95 per cent.[2] The general cold douche is a useful adjunct in the treatment of *obesity*. Very hot local douches relieve the pain of *lumbago*, *muscular rheumatism*, and *subacute arthritis*. Cool or cold ones are prescribed as a tonic remedy in later stages of the treatment of inflammatory affections, and are of great service in combination with massage in the treatment of old *fractures* and injuries and in *chronic synovitis*. The Aix douche is indicated in all *toxæmic* or *lithæmic* conditions, especially, of course, when

[1] *Centralbl. f. klin. Med.*, 1893, xiv. 177. [2] *Deutsche med. Wchnschr.*, Dec. 27, 1894.

characterised by a *high tension pulse*, as in so-called "*suppressed gout*," and also relieves some cases of *chronic rheumatism*.

Ascending douches of low or graduated temperature promote peristalsis, and are therefore indicated in atonic *constipation* and in some cases of *piles*. More painful and *acute hæmorrhoidal* symptoms and *pruritus* are often greatly relieved by the ascending spray of a temperature as high as is bearable.

Fig. 4. CONTINUOUS CURRENT BATH.
SHOWING BATTERY, CELL-COLLECTOR, SWITCH-BOARD, GALVANOMETER, AND PEDAL ELECTRODE.
ALSO SHOWS TRANSFORMER FOR CURRENT FROM THE MAINS.

The above are representative examples of a few of the more common hydro-thermal methods, but space will not permit of a description of others of equal utility. A second section might be devoted to packs, fomentations, &c., and a third to the various *immersion* baths (hot, cold, indifferent, saline, and medicated), including the well-known *Nauheim* treatment of heart-disease; but their discussion is necessarily omitted at present.

### III. Electric and Hydro-Electric.

The forms of electricity commonly employed are (1) *Galvanic* and (2) *alternating* or *induced*, the source of supply being, in the first case, a battery of twenty-four large Leclanché cells, regulated by a cell collector, milliampère meter, and graphite rheostat (Fig. 4), and employed both for local and general dry galvanisation and for continuous current baths; and, in the second case, (*a*) an ordinary dry-cell chloride of silver Faradic battery for dry electricity and Farado-massage, and (*b*) for baths, the current from the public electric-lighting circuit. The method of utilising dynamo currents with safety and convenience for medical purposes has been well described by Dr. W. S. Hedley in his work *Current from the Main*, and need not be repeated here. The Bristol authorities supply their current at a pressure of 105 volts, and with alternations at the rate of 93 complete periods per second. To avail ourselves of this, an ordinary incandescent lamp is replaced by a plug, or adapter, and the current so obtained is led through a transformer (Fig. 4), by means of which the voltage is reduced to suitable dimensions and regulated at will. The result is a current resembling somewhat the ordinary Faradic current, but of a less harshly pulsatile, more wavy and uniform character, being produced by an electromotive force of which the positive and negative maxima are approximately equal, and attained by smooth, though rapid, transitions. Such a current is described by Hedley as being "more or less *sinusoidal*," and is represented by a "curve of sines." It is pleasant to the patient, and is well adapted for use in the class of cases for which Faradic treatment is prescribed, having probably for therapeutic purposes (especially for use in connection with baths) some advantages over the latter form of electricity.

*Forms of Electrical Treatment:*

(*a*) Dry electricity and electro-massage.

i. *Galvanisation.* Weak currents, ascending or descending as prescribed, and of approximately stated strength,[1] are applied for a certain period to (1) the whole or (2) affected parts of the body.

ii. *Faradisation*, general or local, to the part specified, and during the prescribed time.

ii. *Farado-massage.* One electrode is in contact with the patient's body (usually the dorsal spine), the other is formed by a metal clasp worn on the right wrist of the operator. For prescribed period, general or local.

[1] Say 3 to 20 milliampères registered while the current is actually passing through the part under treatment.

(*b*) Electric baths.

i. *Galvanic.* General (Fig. 4). Temperature of water, 98° F. The patient reclines at full length on his back, the body just immersed. The direction of the current is usually from the feet towards the head. A slight pricking sensation may or may not be felt at the ankles and knees, or a sensation of warmth near the negative electrode. The current is usually uninterrupted, and of a strength not exceeding 100 milliampères at the first bath, but gradually raised to 200 or 250 by, say, the third or fourth bath. Duration, 15 to 20 minutes.

ii. *Alternating (sinusoidal) electric bath.* Administer as above, but with the alternating current from the main in circuit, modified by a suitable transformer. The strength should be gradually increased, short of the point at which contraction of the muscles is induced, and then gradually diminished during the last two minutes of the bath. Duration, 20 minutes.

iii. *Combined Galvanic and sinusoidal bath.* With both currents simultaneously passed along the wires; the galvanic current not, as a rule, to exceed 100 milliampères; the alternating current graduated as in ii. Duration, 20 minutes.

iv. *Electric footbath (sinusoidal).* The feet, immersed in hot water, rest on a large flat electrode at the bottom of an insulated footbath. The other electrode, consisting of a large damp sponge, is held in both hands. After two minutes the strength of the current is, by means of the transformer, gradually increased, and then brought back to its original degree. Duration, 10 minutes.

*Physiological Effects and Indications.*—The effects of the bipolar electric bath are thus summed up by Erb:[1] Respiration diminished, metabolism and renal secretion decidedly increased. Appetite and digestion are improved; the genital functions are stimulated; circulation and nutrition are benefited; sleep notably restored, and new vigour imparted to the mental and physical faculties. While recognising the accuracy of this general statement, Dr. Hedley points out that a good deal has been learned about the electric bath since this was written. Those interested in the subject may be referred to his excellent work, *Hydro-Electric Methods.*

[1] *Electrotherapeutics*, 1887, p. 280.

It is superfluous to discuss in detail the indications for ordinary Faradic and Galvanic treatment of chronic diseases; *e.g.*, *motor*, *sensory*, and *trophic affections* of the neuro-muscular system, &c. Dr. D. Graham reports excellent results from the treatment of *acute gout* by massage in combination with brief Galvanisation of the inflamed joints. A current of 5 to 10 milliampères was employed for a few minutes, the cathode being applied to the affected region. There was evident diminution of the congestion, induration, and œdema. Farado-massage is often serviceable in *neurasthenia*, *hysteria*, and general *malnutrition*. The Galvanic bath has a wide field of therapeutic utility. Hedley reports excellent results from its employment in cases of *insomnia*, *muscular rheumatism*, and *neurasthenia*, enhanced in the case of the last-named by Galvanisation of the head. The *gouty state* is benefited by a course of such baths, given in the intervals between attacks, and in selected cases of *locomotor ataxy* symptoms are alleviated by its use. I can support, from my own observations, the claims of Hedley, Armstrong, Nevison, and others for the continuous current bath that it is, perhaps, the best single remedy known for cases of *rheumatoid arthritis*, especially those of the neurotic or paretic type. The bath seems to supply the needful combination of central and peripheral treatment. Apart from the reduction of local manifestations, the general health is stimulated, and a feeling of warmth and comfort lasting for the remainder of the day is a noticeable effect of individual baths. Chauvet,[1] of Royat, obtained satisfactory results by the hydro-galvanic treatment in ten out of fourteen cases of rheumatoid arthritis, though in two of these the baths had to be discontinued owing to the supervention of nervous excitability. There was a remarkable and rapid improvement in the mobility of the joints. Still better results might be expected by the alternate employment of the continuous current bath and the radiant heat treatment, described above.

Somewhat different are the indications for the use of the alternating (sinusoidal) electric bath. By this form of vibratory treatment the irritability of hypersensitive nerves may be

[1] *Arch. d'Électric. méd.*, 1898, vi. 151.

painlessly diminished. Hence, in part, their proved efficacy in many forms of *neuritis* and neuralgic affections. Hedley reports the absolute relief of a case of *trigeminal neuralgia*. Of six cases of simple *sciatica* treated at St. Bartholomew's with the sinusoidal bath, Dr. Percy Lewis cured three and relieved two others. Gautier and Larat report the successful treatment of *chlorosis*, *spinal neurasthenia*, *struma*, *rachitis*, *muscular atrophies*, *torticollis*, and *lumbago*, and the permanent cure of some cases of *obesity*. Cutaneous neuroses (*e.g.*, *urticaria*) are also sometimes amenable to this method, and the same authors report the cure by this means of a case of *eczema* of thirty years' standing.[1]

The electric footbath is a useful adjunct in the treatment of many forms of nervous *debility*. Given in the latter part of the day, it tends to promote sleep in cases of *insomnia*. Dr. Lewis, while employing it at the Hospital for cases of *infantile paralysis*, made the useful discovery that a short course of electric foot-baths is an effectual remedy for *chilblains*.

The combined Galvanic and sinusoidal general electric bath may be tried in cases where the indications for Galvanic and alternating currents clearly coincide (as may often be the case in rheumatoid arthritis, rheumatism complicated by neuralgias, &c.), or where, while electrical treatment is obviously called for, both forms employed singly fail to produce the expected result.

---

### RHEUMATIC DISEASE OF THE CARDIAC MUSCLE.

BY

THEODORE FISHER, M.D.Lond., M.R.C.P.,

*Pathologist to the Bristol Royal Infirmary, Physician to Out-Patients to the Bristol Royal Hospital for Sick Children and Women.*

---

ALL text-books on medicine speak of rheumatism as one of the causes of myocarditis, but the simple statement usually stands alone without comment. Clinically, the possibility of lesions of

[1] *Hydro-Electric Methods*, by W. S. Hedley, 2nd Ed., 1896.

## Figures and Tables

**Fig. 1. f1:**
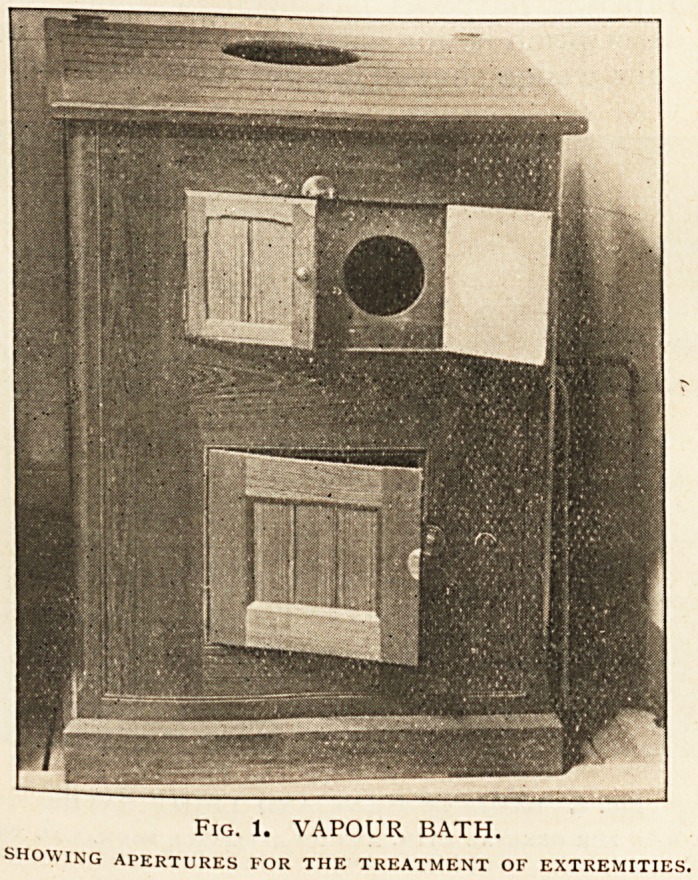


**Fig. 2. f2:**
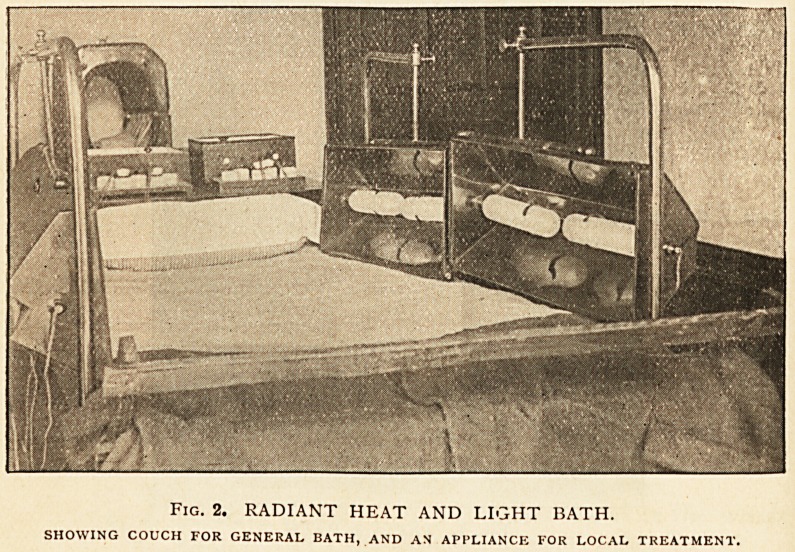


**Fig. 3. f3:**
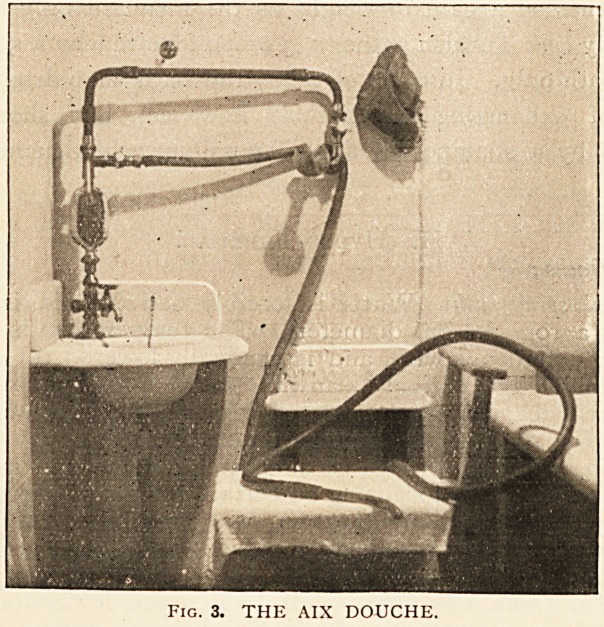


**Fig. 4. f4:**